# Fucoxanthin Ameliorates Carbon Tetrachloride‐Induced Liver Fibrosis in Mice via Nrf2/HO‐1/GPX4‐Mediated Ferroptosis Pathway

**DOI:** 10.1002/fsn3.70589

**Published:** 2025-07-10

**Authors:** Zhongliang Liu, Jiena Ye, Jiachen Xi, Qingping Li, Yunping Tang, Yizhou Tian, Dongxu Wang, Zuisu Yang, Yaping Ding

**Affiliations:** ^1^ Zhoushan Hospital of Traditional Chinese Medicine Affiliated to Zhejiang Chinese Medical University Zhoushan China; ^2^ School of Food and Pharmacy Zhejiang Ocean University Zhoushan China; ^3^ Zhejiang Chinese Medical University Hangzhou China; ^4^ School of Grain Science and Technology Jiangsu University of Science and Technology Zhenjiang China

**Keywords:** ferroptosis, fucoxanthin, glutathione peroxidase 4, liver fibrosis, Nrf2

## Abstract

Liver fibrosis, closely linked to oxidative stress, remains a significant global health challenge. Fucoxanthin (Fx), a marine carotenoid extracted from brown algae, exhibits potent antioxidant properties, yet its molecular mechanism in liver fibrosis remains unclear. In the present study, a murine model of liver fibrosis was established through intraperitoneal injection of carbon tetrachloride (CCl_4_) to investigate the therapeutic potential and underlying mechanisms of Fx. Histological staining and transmission electron microscopy were utilized to evaluate liver morphology, while assessments were conducted on hepatic function indicators, antioxidant indices, liver fibrosis markers, and inflammatory factors. Notably, treatment with Fx resulted in a significant improvement in serum liver function indicators compared to CCl_4_ model mice. Furthermore, the levels of liver fibrosis markers and inflammatory factors were significantly decreased following Fx treatment. Moreover, Fx treatment led to a significant downregulation of hydroxyproline and alpha‐smooth muscle actin (α‐SMA) expression, while upregulating key antioxidative and ferroptosis‐related proteins. These proteins include nuclear factor erythroid 2‐related factor 2 (Nrf2), heme oxygenase‐1 (HO‐1), NAD(P)H:quinone oxidoreductase 1 (NQO1), glutamate–cysteine ligase modifier (GCLM), glutathione peroxidase 4 (GPX4), solute carrier family 7 member 11 (SLC7A11), transferrin receptor 1 (TFR1), and ferritin light chain (FTL) protein expression, in comparison to the CCl_4_ model mice. These findings suggest that Fx could effectively ameliorate liver fibrosis by mitigating CCl_4_‐induced oxidative stress and ferroptosis, highlighting its therapeutic potential in liver fibrosis management.

## Graphical abstract

Fucoxanthin can ameliorate CCl_4_‐induced liver fibrosis in mice. Fucoxanthin can mitigate hepatic oxidative stress and ferroptosis in mice. Modulation of the Nrf2/HO‐1/GPX4 pathway by fucoxanthin may contribute to the protective effect.

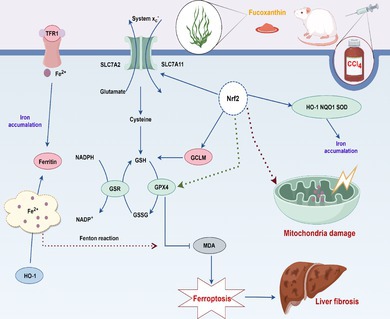

**Abbreviations**
α‐SMA: alpha‐smooth muscle actin
AKP: alkaline phosphatase
ALB: albumin
ALT: alanine aminotransferase
AST: aspartate aminotransferase
CAT: catalase
CCl_4_
: carbon tetrachloride
Col IV: type IV collagen
ELISA: enzyme‐linked immunosorbent assay
ER: endoplasmic reticulum
FTL: ferritin light chain
Fx: fucoxanthin
GCLM: glutamate‐cysteine ligase modifier subunit
GPX4: glutathione peroxidase 4
GSH‐Px: glutathione peroxidase
GSH‐ST: glutathione‐*S* transferase
HA: hyaluronic acid
HE: hematoxylin and eosin
HO‐1: heme oxygenase‐1
HYP: hydroxyproline
IHC: immunohistochemistry
IL‐1β: interleukin‐1 beta
IL‐6: interleukin‐6
LN: laminin
MDA: malondialdehyde
NQO1: NAD(P)H:quinone oxidoreductase 1
Nrf2: nuclear factor erythroid 2‐related factor 2
PC3: type III procollagen
SLC7A11: solute carrier family 7 member 11
SOD: superoxide dismutase
T‐AOC: total antioxidant capacity
T‐BIL: total bilirubin
TFR1: transferrin receptor 1
TNF‐α: tumor necrosis factor‐alpha

## 1 Introduction

The liver plays an essential metabolic role in the human body, including the detoxification of hazardous substances; metabolism of proteins, lipids, carbohydrates, and vitamins; production of steroids; and secretion of bile, to support the functions of other organs (Wunderlich et al. 2014). Liver fibrosis, caused by activated fibroblasts and abnormal connective tissue proliferation, is a wound‐healing response (Lee et al. 2015; Yang et al. 2021). Various factors, including alcohol consumption, viral infection, chronic hepatitis, obesity, and immune system imbalance, can lead to the development of liver fibrosis (Ganai and Husain 2017). Regardless of the cause, liver fibrosis usually results from chronic liver injury and can progress to cirrhosis (Yang et al. 2017; Wang, Huang, et al. 2020). Reversing or delaying liver fibrosis can effectively reduce the incidence of liver failure; however, no effective antifibrosis drugs exist. Therefore, identifying nontoxic and effective compounds for liver fibrosis treatment has far‐reaching significance and broad prospects.

Algae, as the most abundant and diverse marine plants, are part of the lower cryptogams of the ocean. To date, hundreds of structurally novel carotenoids have been identified from algae, with being one of the most prominent. Fucoxanthin (Fx) has garnered increasing attention for its broad spectrum of bioactive properties, including antioxidant, antibacterial, anti‐inflammatory, anticancer, antidiabetic, antihypertensive, antihyperlipidemic, anti‐obesity, and hepatoprotective effects (Xiao et al. 2020; Das et al. 2022). Previous studies conducted in our laboratory have shown that the protective effects of Fx against acute alcoholic liver injury in mice are associated with the activation of nuclear factor erythroid 2‐related factor 2 (Nrf2)‐mediated antioxidant defenses and the suppression of Toll‐like receptor 4 (TLR4)‐mediated inflammatory responses (Zheng et al. 2019). Fx has been shown to prevent organ fibrosis (Guan et al. 2022; Cui et al. 2023). In a study, dietary Fx inhibited hepatic oxidative stress, thereby preventing the diet‐induced early phase of fibrosis in mice (Takatani et al. 2020). Additionally, Fx's antifibrotic effects have been demonstrated in pulmonary fibrosis, where it significantly attenuates collagen deposition in the lung (Ma et al. 2017). Fx inhibits the expression of profibrotic genes, further confirming its antifibrotic potential in vitro and in vivo (Kim et al. 2019; Slautin et al. 2024). In addition, Fx has been shown to reduce the expression of inflammation‐related factors and protected against zearalenone‐induced liver injury as well as inflammatory responses (Li et al. 2020; Ben et al. 2023). Beyond its antioxidant and hepatoprotective effects, Fx exhibits a broad range of physiological and biological abilities, such as anti‐obesity, antitumor, and antidiabetic properties (Din et al. 2022). However, its role in inhibiting murine liver fibrosis and the underlying mechanisms have yet to be fully elucidated.

Ferroptosis, a nonapoptotic form of cell death characterized by the iron‐dependent production of lipid peroxides, is initiated by glutathione peroxidase 4 (GPX4) activity attenuation and lipid oxidation (Qi et al. 2020; Wang, Liu, et al. 2020). Bai et al. demonstrated that obacunone attenuated liver fibrosis by enhancing GPX4 protein expression (Bai et al. 2021). Recent studies have revealed that GPX4‐deficient mice exhibited hepatocyte degeneration, leading to premature mouse death and indicating that GPX4 protected liver function from harmful lipid peroxidation (Carlson et al. 2016). In addition, GPX4 inactivation stimulated ferroptosis, which ultimately caused ethanol‐induced hepatocyte death (Luo et al. 2023). The above results indicate a close relationship among lipid peroxidation, GPX4, and ferroptosis. Nrf2, as a central controller of multiple antioxidant genes, is instrumental in shielding cells against oxidative stress (Song and Long 2020; Dodson et al. 2019; Soula et al. 2020). In addition to regulating antioxidant genes, it regulates almost all ferroptosis‐related genes (Gao et al. 2014). Although Nrf2 promotes ferroptosis sensitivity, its activation leads to ferroptosis resistance in cancer cells (Kerins and Ooi 2018; Lee 2017). Moreover, research has demonstrated that fibrosis is exacerbated in mice after Nrf2 or GPX4 loss, indicating the essential relationship of Nrf2 with ferroptosis (Tsubouchi et al. 2019).

The toxic effects of carbon tetrachloride (CCl_4_) involve oxidative stress and free radical generation, which induce excessive reactive oxygen species (ROS) production, promote iron accumulation, and trigger lipid peroxidation, all of which are key events in ferroptosis (Dai et al. 2021). Studies have demonstrated that ferroptosis contributes to the activation of hepatic stellate cells via mechanisms such as ROS accumulation, lipid peroxidation, and glutathione depletion, thereby promoting fibrogenesis (Cho et al. 2022). Ferroptosis may play an important role in the onset and progression of a variety of fibrotic diseases, especially in hepatic fibrosis, pulmonary fibrosis, renal fibrosis, and other diseases (Pei et al. 2025). Given the potential role of ferroptosis in fibrosis, modulation of iron metabolism or inhibition of ferroptosis may become a novel strategy for the treatment of fibrosis.

We and other previous studies have shown that Fx can modulate ferroptosis by influencing iron homeostasis and regulating ferroptosis‐related proteins such as GPX4 (Du et al. 2024; Ding et al. 2025). In the present study, an animal model was established through the injection of CCl_4_, and the ameliorative effect of Fx was explored. Fx treatment significantly ameliorated fibrosis, with its underlying mechanisms linked to the activation of Nrf2 signaling and the inhibition of GPX4‐mediated ferroptosis. These findings provide new insights and a theoretical foundation for the potential application of Fx in preventing or treating liver fibrosis resulting from chemical liver injury. The present study aimed to demonstrate the therapeutic effect of Fx on CCl_4_‐induced hepatic fibrosis through the Nrf2/HO‐1/GPX4‐mediated ferroptosis pathway in vivo.

## 2 Materials and Methods

### 2.1 Animals and Treatment

**FIGURE 1:**
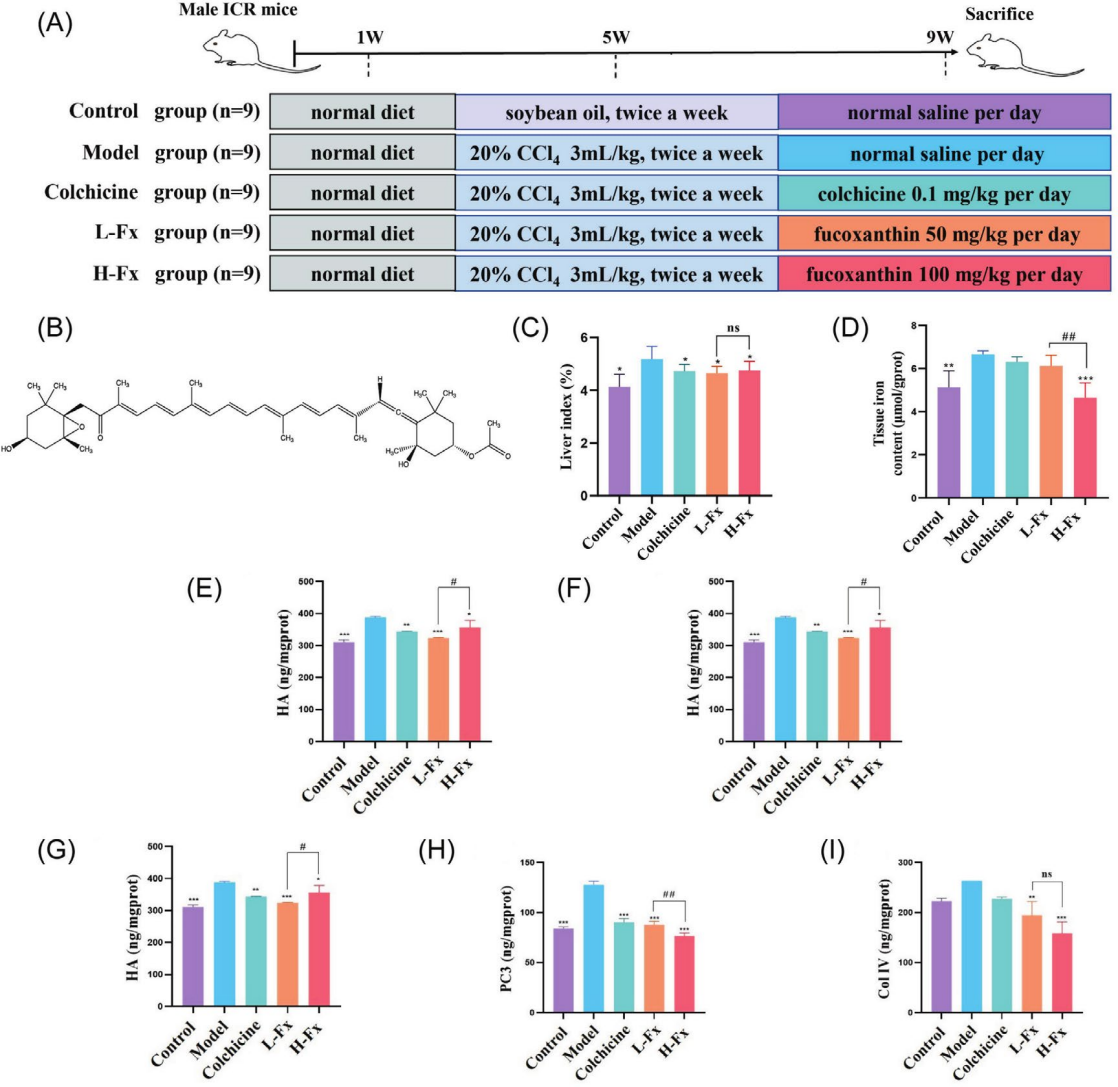
Experimental schedule, and the effects of on liver index, iron level, and degree of fibrosis in the liver of CCl_4_‐induced mice. (A) Experimental schedule. (B) Chemical structure of Fx. (B) Experimental schedule. (C) Liver index (liver weight/body weight). (D) Iron level of liver tissue. (E) Hydroxyproline. (F) Hyaluronic acid (HA). (G) Laminin (LN). (H) Type III procollagen (PC3). (I) Type IV collagen (Col IV). Data are expressed as the mean ± SD (*n* = 9). *p < 0.05, **p < 0.01, and ***p < 0.001 versus CCl_4_ group. ns, Ot significant, ^##^ *p* < 0.01 and ^#^ *p* < 0.05 between the L‐Fx and H‐Fx groups.

Male ICR mice (aged 6–8 weeks and weighing 20 ± 2 g) were procured from the Experiment Animal Center of Zhejiang Province (Hangzhou, China). This study was approved by the Experimental Animal Ethics Committee of Zhejiang Ocean University (Approval No. 2020028). 45 ICR mice were classified into 5 treatment groups, each group containing 9 individuals: control group, model (CCl_4_) group, positive control colchicine (Sigma Aldrich, St. Louis, MO, USA) group, and two Fx (CAS: 3351‐86‐8, Sigma Aldrich, St. Louis, MO, USA, 50 and 100 mg/kg) treatment groups (Figure 1A). This dose is supported by previous in vivo studies showing its therapeutic efficacy and safety (Mao et al. 2022; Yang et al. 2020; Beppu et al. 2009; Lin et al. 2016).

Mice were acclimatized for 1 week in a pathogen‐free animal facility, where they were housed in groups and given unrestricted access to food and water. The facility maintained a controlled temperature range of 20°C–24°C, with relative humidity between 40% and 60%, and adhered to a 12‐h light/dark cycle. From Weeks 2–5, the control group received intraperitoneal injections (i.p.) of soybean oil, whereas the other groups were injected with 20% CCl_4_ (CCl_4_: soybean oil, 1:4, 3 mL/kg, twice a week, i.p.). During the experiment, mice were co‐housed with two to five mice per cage. From Weeks 6–9, oral administration of 0.1 mL of 0.9% NaCl per 10 g of mouse body weight once a day was carried out in both the control and CCl_4_ groups. In the positive control group, colchicine was orally administered at a daily dose of 0.1 mg/kg, with a volume of 0.1 mL/10 g body weight. Similarly, the L‐Fx (50 mg/kg) and H‐Fx (100 mg/kg) groups received a daily oral dose of Fx, with a volume of 0.1 mL/10 g body weight for 4 weeks. Fx was first dissolved in a small amount of dimethyl sulfoxide and then diluted with 0.9% NaCl to the target concentration. The solution was freshly prepared each day, protected from light, and thoroughly mixed to ensure clarity. At the end of Week 9, 24 h after the final treatment, all mice were fasted and then sacrificed for sample collection. To ensure humane handling, all animals were first anesthetized using 3% isoflurane inhalation until deep anesthesia was achieved. Cervical dislocation was then performed for euthanasia.

### 2.2 Sample Collection and Processing

While under deep anesthesia with 3% isoflurane, mice were subjected to retro‐orbital blood collection. The amount of blood collected varied depending on the individual mouse, typically ranging from 0.5 to 1.0 mL. Blood samples were placed in non‐anticoagulant tubes and allowed to clot at room temperature for 30 min. The samples were then centrifuged at 3000 rpm for 10 min at 4°C, and the supernatant serum was collected. The serum was aliquoted and stored at −80°C for subsequent biochemical and cytokine analyses.

### 2.3 Measurement of Iron Level in Liver Tissues

The liver of mice was mixed with normal saline and ground on ice to obtain a liver tissue homogenate. Then, the iron level in the tissue homogenate was detected as per kit instructions (A039‐2‐1, Jiancheng Co., Nanjing, China).

### 2.4 Biochemical Analysis

For serum biochemical analyses, the collected blood specimens were centrifuged at 7140 *g* at a temperature of 4°C for 10 min. Alanine aminotransferase (ALT, C009‐2‐1), aspartate aminotransferase (AST, C010‐2‐1), alkaline phosphatase (AKP, A059‐2‐2), glutathione‐*S*‐transferase (GSH‐ST, A004‐1‐1), albumin (ALB, A028‐2‐1), and total bilirubin (T‐BIL, C019‐1‐1) levels were measured using their corresponding kits (Jiancheng Co., Nanjing, China) as a reflection of liver function. Furthermore, a portion of liver tissue weighing 0.2 g was combined with 1.8 mL saline in an ice‐cold environment to prepare a tissue homogenate. The protein concentration of each sample was determined using the BCA protein assay kit (Solarbio, Beijing, China) according to the manufacturer's instructions. All biochemical markers in liver tissue were normalized to milligrams of total protein.

The lipid peroxidation level and antioxidative capacity of the hepatic homogenate were assessed via glutathione peroxidase (GSH‐Px, A005‐1‐2), malondialdehyde (MDA, A003‐1‐2), superoxide dismutase (SOD, A001‐2‐1), GSH‐ST, total antioxidant capacity (T‐AOC, A015‐3‐1), and catalase (CAT, A007‐1‐1) levels using appropriate kits (Jiancheng Co., Nanjing, China).

### 2.5 Measurement of Hepatic Inflammatory Cytokines

In the liver tissue homogenate samples, the level of several inflammatory cytokines, including interleukin‐6 (IL‐6, KE10007), tumor necrosis factor‐alpha (TNF‐α, KE10002), and interleukin‐1 beta (IL‐1β, KE10003), was assessed via enzyme‐linked immunosorbent assay (ELISA) kits obtained from Proteintech Group Inc. (Wuhan, China). The expression was quantified by establishing a standard curve, and all experiments were performed in triplicate to ensure reliability and precision.

### 2.6 Measurement of Liver Fibrosis Marker

Fresh liver tissue was first hydrolyzed at a high temperature, followed by the estimation of the hydroxyproline (HYP, A030‐2‐1) (Jiancheng Co., Nanjing, China) level as per the manufacturer's instructions. ELISA kits procured from Jiancheng Co. (Nanjing, China) were used to measure hyaluronic acid (HA, H141‐1‐2), laminin (LN, H148‐1‐2), type III procollagen (PC3, H212), and type IV collagen (Col IV, H145‐1‐2) levels in mouse liver tissue.

### 2.7 Histopathological Analysis

The liver tissue of mice was dissected into 1‐cm^3^ pieces and fixed for 24 h in 4% paraformaldehyde. Following fixation, the liver tissue was subjected to gradient dehydration to remove moisture and increase permeability. The paraffin sections of the dehydrated tissue were stained with hematoxylin and eosin (H&E) and were scored using the Ishak score system to assess liver pathology. The sections were subsequently dewaxed, stained for 8 min in a Sirius red staining solution prepared from saturated picric acid, and finally rinsed with absolute alcohol for several minutes. The Sirius red solution stains collagen fibers in the tissue. The Sirius red‐positive regions were quantified across various fields for each section using ImageJ Software, following the formula: (collagen area/total area—vascular lumen area) × 100.

### 2.8 Transmission Electron Microscopy

The liver tissue of mice was cut into 0.5‐cm^3^ pieces and immediately immersed in 2.5% glutaraldehyde solution for fixation, followed by fixation with 1% osmic acid solution. The fixed liver tissue was gradient‐dehydrated, permeabilized, and immersed in Spurr. The tissues were then cut into 70‐nm thick sections, stained with alkaline lead citrate and uranyl acetate for 5 min each, and finally observed and photographed using a Hitachi H‐7650 transmission electron microscope.

### 2.9 Immunohistochemistry (IHC)

IHC was performed for the detection of Nrf2 and GPX4 in the tissue sections. The sections were subjected to standard processing as per the manufacturer's guidelines. In brief, the sections were incubated with Nrf2 (16396‐1‐AP, 1: 200) and GPX4 (14432‐1‐AP, 1: 200) antibodies (Proteintech Inc., Princeton, NJ, USA) at 4°C for 12 h, followed by incubation with the first biotinylated secondary antibody (1:100) for 60 min and then SABC (1: 100) for 30 min at 37°C. Then, DAB color development was followed by gradient dehydration and blocking with xylene. Finally, images were captured under a light microscope and analyzed semi quantitatively using the ImageJ software.

### 2.10 Western Blotting

Lliver tissue was ground with liquid nitrogen to obtain a powder. Proteins were extracted from the powder using radioimmunoprecipitation assay buffer and processed via electrophoresis, polyvinylidene fluoride membrane transfer, and blocking. The following primary antibodies were added to the polyvinylidene fluoride membranes: Nrf2 (1: 2000), GPX4 (1:1000), alpha‐smooth muscle actin (α‐SMA) (14395‐1‐AP, 1:2000), solute carrier family 7 member 11 (SLC7A11) (26864‐1‐AP, 1:2000), NAD(P)H:quinone oxidoreductase 1 (NQO1) (67240‐1‐Ig, 1:2000), transferrin receptor 1 (TFR1) (10084‐2‐AP, 1:2000), glutamate‐cysteine ligase modifier subunit (GCLM) (14241‐1‐AP, 1:2000), ferritin light chain (FTL) (10727‐1‐AP, 1:2000), and heme oxygenase‐1 (HO‐1) (66743‐1‐Ig, 1:2000) (Proteintech Inc., Princeton, NJ, USA). The membranes were subsequently incubated with a secondary antibody (1:1000) and detected and analyzed using the Alpha View software.

### 2.11 Statistical Analysis

All values are expressed as mean ± standard deviation. Statistical analyses were conducted using GraphPad Prism version 8.3.0 (GraphPad Software Inc., La Jolla, CA). The Shapiro–Wilk test was employed to assess data normality. For normally distributed data, differences between two groups were evaluated using one‐way analysis of variance (ANOVA), with Tukey's post hoc test applied for multiple comparisons. Non‐normally distributed data were analyzed using the Mann–Whitney *U* test for two independent samples and Kruskal–Wallis test and Nemenyi test for multiple independent samples. *p* < 0.05 was considered statistically significant.

## 3 Results

### 3.1 Effect of Fx on Liver Index and Tissue Iron Level in CCl_4_ ‐Induced Mice

In the control group, the liver index was 4.13% ± 0.4 7%. However, it increased significantly to 5.18% ± 0.47% (*p* < 0.05) in the CCl_4_ group. By contrast, the liver index of L‐Fx and H‐Fx groups decreased to 4.65% ± 0.25% and 4.75% ± 0.35%, respectively, which were significantly different from that of the CCl_4_ group (*p* < 0.05). However, there was no significant difference between L‐Fx and H‐Fx with regard to their effect on the liver index (Figure 1C). The iron level in the liver tissue was 5.13 ± 0.77 μmol/g protein in the control group and increased significantly to 6.66 ± 0.16 μmol/g protein after CCl_4_ treatment (*p* < 0.05). However, the H‐Fx group significantly reduced the iron level to 4.65 ± 0.69 μmol/g protein (*p* < 0.001) (Figure 1D).

### 3.2 Fx Ameliorated Degree of Fibrosis in CCl_4_ ‐Induced Mice

In the present study, we found that major liver fibrotic biomarkers (HYP, HA, LN, PC3, and Col IV) were notably elevated in the CCl_4_ group (*p* < 0.001). By contrast, Fx treatment significantly downregulated these hepatic fibrotic marker levels in CCl_4_‐induced mice (*p* < 0.001). These findings indicated that Fx improved liver fibrosis by decreasing levels of key fibrotic markers.

### 3.3 Effect of Fx on Liver Function in Mice

**FIGURE 2:**
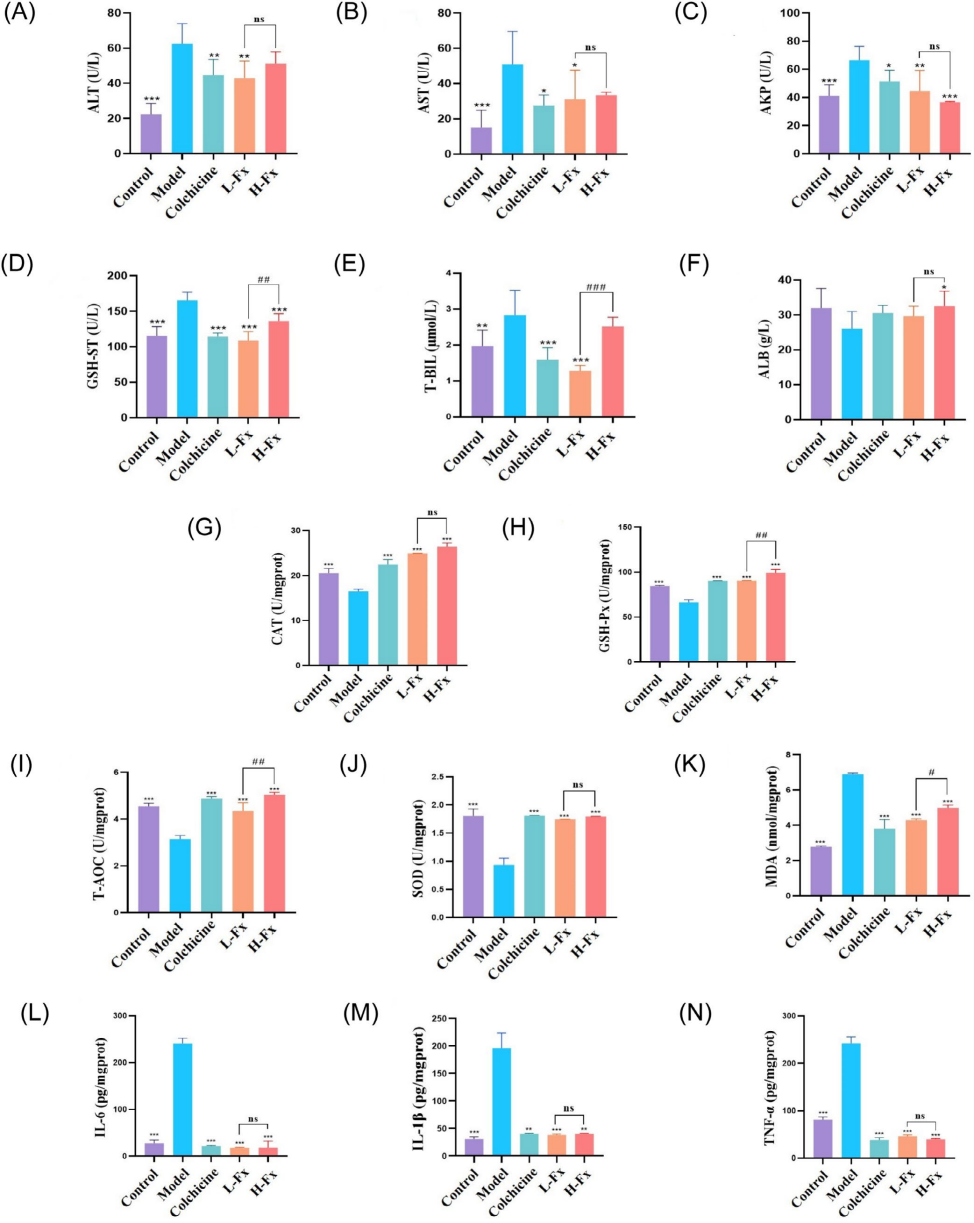
Effect of Fx on liver function, antioxidant capacity, and inflammatory response of CCl_4_‐induced mice. (A) Serum alanine aminotransferase (ALT) level. (B) Serum aspartate aminotransferase (AST) level. (C) Serum alkaline phosphatase (AKP) level. (D) Serum glutathione‐*S* transferase (GSH‐ST) activity. (E) Serum total bilirubin (T‐BIL) level. (F) Serum albumin (ALB) level. (G) Hepatic catalase (CAT) activity. (H) Hepatic GSH‐ST activity. (I) Hepatic total antioxidant capacity (T‐AOC) content. (J) Hepatic superoxide dismutase (SOD) activity. (K) Hepatic malondialdehyde (MDA) content. (L) Hepatic interleukin‐6 (IL‐6) level. (M) Hepatic interleukin‐1 beta (IL‐1β) level. (N) Hepatic tumor necrosis factor‐alpha (TNF‐α) level. Data are expressed as the mean ± SD (*N* = 9). *p < 0.05, **p < 0.01, and ***p < 0.001 versus CCl_4_ group. ns, Ot significant, ^#^ *p* < 0.05, ^##^ *p* < 0.01, and ^###^ *p* < 0.001 between the L‐Fx and H‐Fx groups.

The serum ALT, AST, and AKP levels were markedly increased in the CCl_4_ group compared with the control group (*p* < 0.001) (Figure 2A–C), indicating liver damage. However, treatment with Fx significantly decreased ALT, AST, and AKP levels, demonstrating that Fx reduced CCl_4_‐induced hepatotoxicity (Figure 2A–C). In addition, serum GSH‐ST and T‐BIL levels were significantly higher in the CCl_4_ group (*p* < 0.01), but treatment with Fx significantly reduced these serum biomarker levels (*p* < 0.001) (Figure 2D,E), indicating that Fx attenuated CCl_4_‐induced hepatotoxicity. Fx also significantly ameliorated the abnormal reduction in serum ALB level, indicating that Fx intervention attenuated CCl_4_‐induced liver injury (Figure 2F). The ALT, AST, AKP, and ALB levels were not significantly different between the L‐Fx and H‐Fx groups, whereas the GSH‐ST and T‐BIL levels were significantly higher in the H‐Fx group (*p* < 0.01).

### 3.4 Effect of Fx on Antioxidant Capacity and MDA Level in Liver Tissue

The CAT, GSH‐Px, T‐AOC, and SOD activity levels were markedly decreased in the CCl_4_ group (*p* < 0.001) (Figure 2G–J), indicating increased oxidative stress in the liver tissues. However, Fx treatment significantly increased these antioxidant parameters in both the L‐Fx and H‐Fx groups, particularly in the H‐Fx group (*p* < 0.001) (Figure 2G–J). Moreover, the level of MDA, a biomarker of lipid peroxidation, was significantly increased in the CCl_4_ group (*p* < 0.001) (Figure 2K). Following Fx treatment, MDA levels were significantly reduced (*p* < 0.001), with the high‐dose Fx (H‐Fx) group showing a greater reduction compared to the low‐dose Fx (L‐Fx) group (*p* < 0.05) (Figure 2K). While CAT and SOD levels did not differ significantly between the L‐Fx and H‐Fx groups, GSH‐Px and T‐AOC levels were significantly elevated in the H‐Fx group (*p* < 0.01).

### 3.5 Effect of Fx on Inflammatory Factors in Liver Tissue

To assess the anti‐inflammatory effect of Fx, several proinflammatory factors were assessed in the liver tissue. As shown in Figure 2L–N, the level of IL‐6, TNF‐α, and IL‐1β in the control group was 27.81 ± 7.21 pg/mg prot, 30.86 ± 4.14 pg/mg prot, and 81.51 ± 5.56 pg/mg prot, respectively. However, the level of these factors was 241.11 ± 11.23 pg/mg prot, 196.15 ± 27.83 pg/mg prot, and 242.19 ± 13.76 pg/mg prot, respectively, in the CCl_4_ group, which was significantly higher (*p* < 0.001). By contrast, Fx intervention resulted in significant reductions to 18.11 ± 1.16 pg/mg prot, 38.23 ± 1.65 pg/mg prot, and 39.97 ± 1.39 pg/mg prot, respectively (*p* < 0.001). However, the effect of Fx in reducing the level of inflammatory factors was not significantly different between the two Fx treatment groups.

### 3.6 Fx Attenuated Liver Injury and Collagen Deposition in CCl_4_ ‐Induced Mice

**FIGURE 3:**
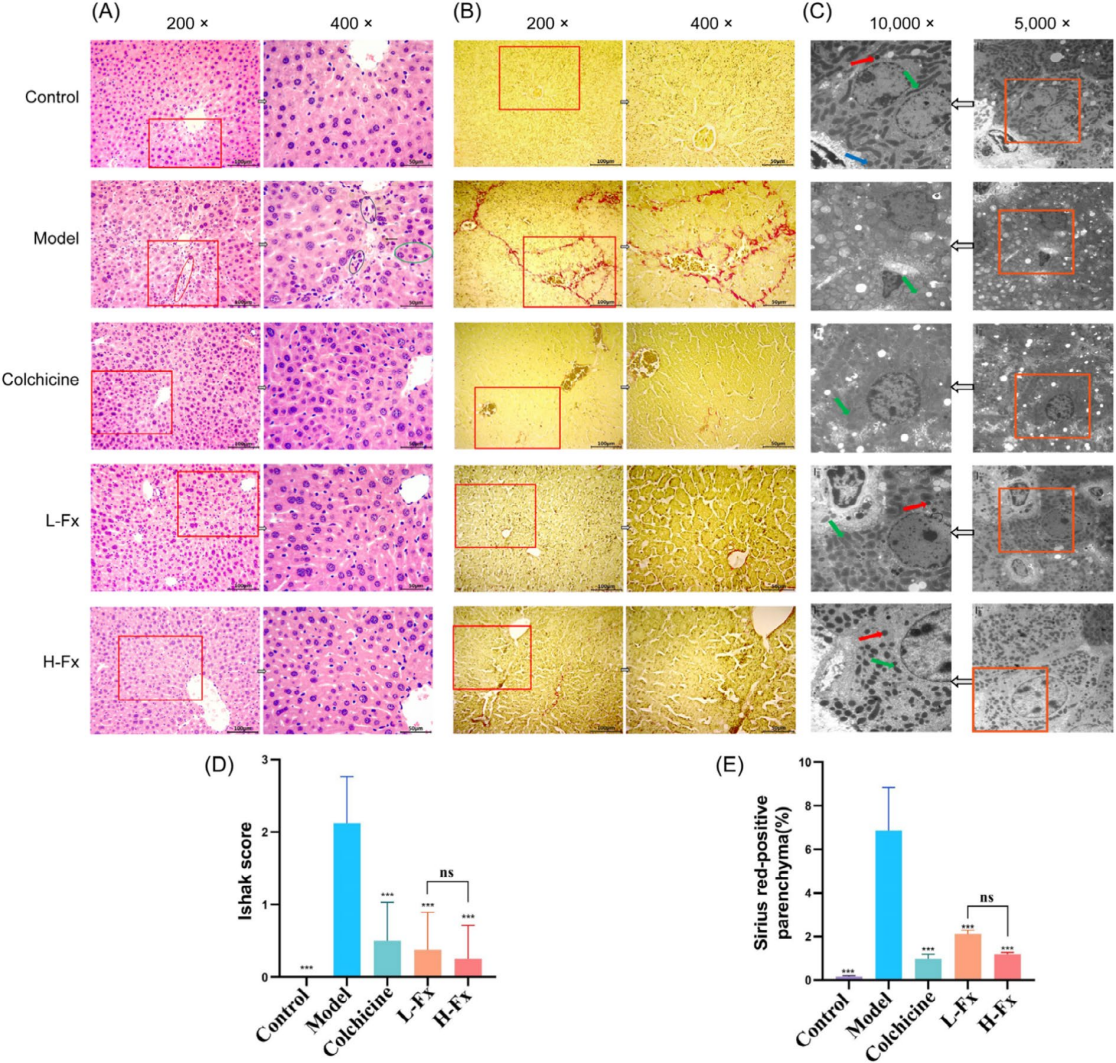
Effect of fucoxanthin (Fx) on the histomorphology and ultrastructure of the liver of CCl_4_‐induced mice. (A) H&E staining (Green circle indicates necrosis; black circle indicates inflammatory infiltration; red circle indicates fibrosis; and black arrow represents iron deposition cell). (B) Sirius red staining. (C) Transmission electron microscopy (Green arrow indicates mitochondria). (D) Histopathological analysis performed using the liver fibrosis Ishak score for each group. (E) Sirius red‐positive parenchyma (%). ×200, scale bar is 100 μm; ×400, scale bar is 50 μm; ×5000, scale bar is 2 μm; ×10,000, scale bar is 1 μm. Data are expressed as the mean ± SD (*n* = 9). ***p < 0.001 vs. CCl_4_ group. ns: Ot significant between the L‐Fx and H‐Fx groups.

Figure 3A shows that the control group's mice liver had a normal lobular structure with central veins and radial hepatic cords. In contrast, the CCl_4_ group exhibited significant interlobular changes, including extensive fibrous tissue growth and pseudolobules. Hepatocytes showed degeneration and necrosis, with pitting and focal necrosis, and some nuclei were missing or enlarged. Additionally, there was heavy infiltration of inflammatory cells in the hyperplastic area, along with fragmentary necrosis. The normal group had an Ishak score of 0, while the CCl_4_ group averaged 2.125, which decreased to below 0.5 after Fx treatment (Figure 3D). Some brown deposits, indicating iron buildup, were found in the CCl_4_ group's liver. Fx significantly reduced CCl_4_‐induced liver fibrosis. Sirius red staining (Figure 3B) showed sparse collagen fibers in the control group but a significant increase in the CCl_4_ group. However, Fx treatment markedly reduced collagen deposition in the liver. In the control group, many mitochondria with rough endoplasmic reticulum (ER) were observed. However, CCl_4_‐treated mice showed fewer mitochondria and rough ER, along with more lysosomes, vacuoles, and lipid droplets. Treatment with Fx significantly improved these conditions (Figure 3C). Sirius red staining showed few collagen fibers in the control group, while the CCl_4_ group had a 6.87% collagen fiber area. In contrast, the colchicine, L‐Fx, and H‐Fx groups reduced this area to 0.97%, 2.12%, and 1.2%, respectively (Figure 3E). The Fx treatments had similar effects on reducing fibrosis. Sirius red staining showed few collagen fibers in the control group, while the CCl_4_ group had a 6.87% collagen fiber area. In contrast, the colchicine, L‐Fx, and H‐Fx groups reduced this area to 0.97%, 2.12%, and 1.2%, respectively (Figure 3E). The effect of Fx in alleviating fibrosis was not significantly different between the two Fx treatment groups.

### 3.7 Fx Increased Expression of Nrf2 and GPX4 Proteins in Liver of CCl_4_ ‐Induced Mice

**FIGURE 4:**
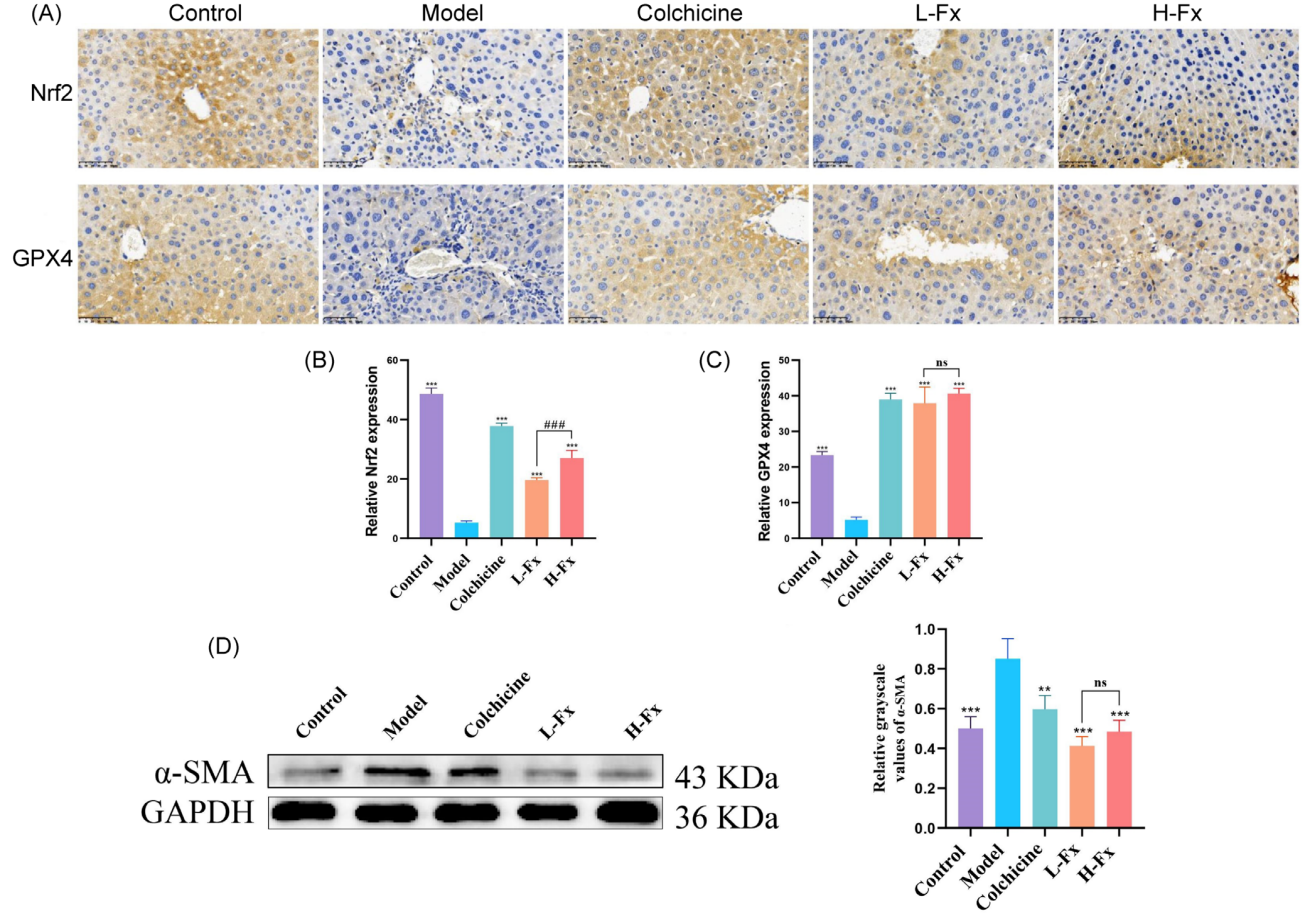
Effect of Fx on the expression of Nrf2, GPX4, and α‐SMA in the liver of CCl_4_‐induced mice. (A) Immunohistochemical analysis of hepatic Nrf2 and GPX4 (×200, scale bar: 100 μm). (B) Quantitative analysis of protein expression of Nrf2; (C) Quantitative analysis of protein expression of GPX4. (D) Hepatic α‐SMA protein expression. Data are expressed as the mean ± SD (*n* = 3). **p < 0.01 and ***p < 0.001 versus CCl_4_ group.: Not significant, ###*p* < 0.001 between the L‐Fx and H‐Fx groups.

The expression levels of Nrf2 and GPX4 were significantly lower or decreased in the CCl_4_ group than in the control group (Figure 4A–C). By contrast, Nrf2 and GPX4 expression levels in the colchicine, L‐Fx, and H‐Fx treatment groups were increased (Figure 4A–C). These results indicate that colchicine and Fx decreased the ferroptosis level. The expression level of Nrf2 was significantly higher in the H‐Fx group than in the L‐Fx group (*p* < 0.001), whereas that of GPX4 was not significantly different between the two Fx dose groups.

### 3.8 Effect of Fx on α‐SMA in CCl_4_ ‐Induced Mice

Liver fibrosis can result from the activation of hepatic stellate cells to produce α‐SMA during liver injury. In the present study, the α‐SMA expression level in the CCl_4_, colchicine, L‐Fx, and H‐Fx groups was 169.92%, 119.19%, 82.41%, and 96.83% of the control group (set at 100%), respectively (Figure 4D), suggesting that colchicine and Fx are effective in treating CCl_4_‐induced liver fibrosis in mice.

### 3.9 Fx Ameliorated Liver Fibrosis by Activating the Nrf2 Pathway in CCl_4_ ‐Induced Mice

**FIGURE 5:**
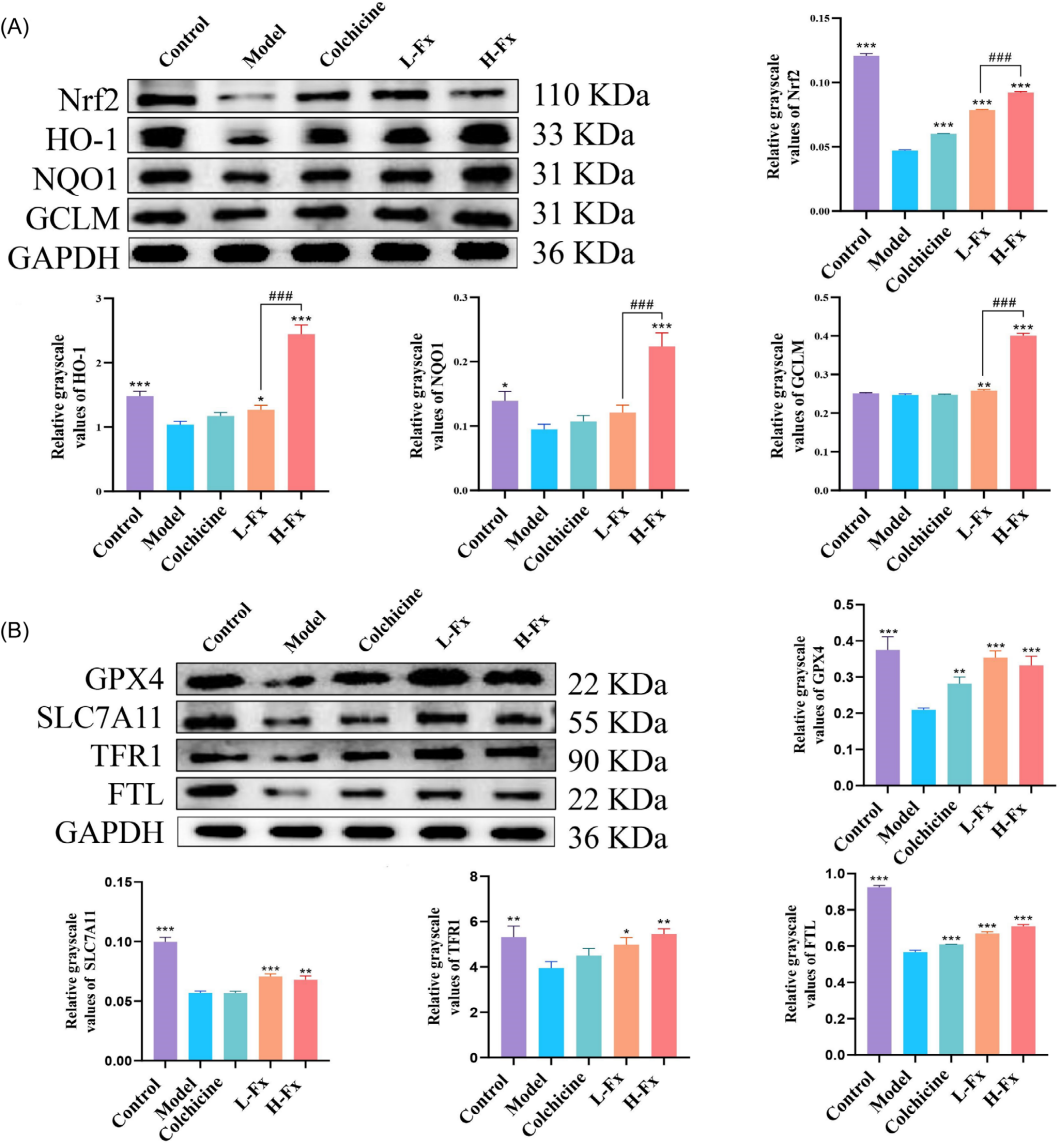
Effect of Fx on Nrf2 and ferroptosis signaling pathways in the liver of CCl_4_‐induced mice. (A) Protein expression of hepatic Nrf2, heme oxygenase‐1 (HO‐1), nicotinamide quinone oxidoreductase 1 (NQO1), and glutamate‐cysteine ligase modifier subunit (GCLM) expression. (B) Protein expression of hepatic GPX4, solute carrier family 7 member 11 (SLC7A11), transferrin receptor 1 (TFR1), and ferritin light chain (FTL). Data are expressed as the mean ± SD (*n* = 3). *p < 0.05, **p < 0.01 and ***p < 0.001 versus CCl_4_ group. ###*p* < 0.001 between the L‐Fx and H‐Fx groups.

Nrf2 is believed to protect against oxidative damage, indicating that CCl_4_‐induced oxidative stress was significantly mitigated by Fx supplementation. Thus, the expression levels of Nrf2 pathway‐related proteins were analyzed to investigate the possible antioxidation mechanism of Fx in liver injury. As shown in Figure 5A, the protein levels of Nrf2 (*p* < 0.001), HO‐1 (*p* < 0.001), NQO1 (*p* < 0.05), and GCLM (*p* > 0.05) were decreased in the CCl_4_ group. However, H‐Fx treatment significantly increased the expression of these Nrf2 pathway‐related proteins. Moreover, the expression levels of Nrf2, HO‐1, NQO1, and GCLM were significantly higher in the H‐Fx group than in the L‐Fx group (*p* < 0.001). These findings demonstrated that Fx produced its effect via the activation of the Nrf2/HO‐1 signaling pathway and that the antioxidant effect was superior in the H‐Fx group.

### 3.10 Fx Ameliorated Liver Fibrosis by Regulating Ferroptosis in CCl_4_ ‐Induced Mice

As shown in Figure 5B, the expression of ferroptosis‐related protein GPX4 and downstream proteins SLC7A11, FTL, and TFR1 in the liver was reduced (*p* < 0.01), following CCl_4_ treatment. However, the expression level of these ferroptosis‐related proteins was significantly increased in the Fx treatment group (*p* < 0.001). These observations imply that Fx may exert a therapeutic effect on liver fibrosis by modulating the process of ferroptosis in mice treated with CCl_4_.

## 4 Discussion

Liver fibrosis is acknowledged as a prevalent manifestation in non‐alcoholic fatty liver disease (NAFLD). Consequently, understanding the molecular mechanisms underlying these conditions and identifying suitable therapeutic agents are crucial for the advancement of potential treatments. Fx has been shown to prevent the progression of liver fibrosis by inhibiting inflammatory processes. Recent clinical studies have further demonstrated that Fx reduces the incidence of NAFLD (Shih et al. 2021). Nevertheless, the precise mechanisms by which Fx mitigates the development and onset of liver fibrosis remain incompletely elucidated. In this study, we found that Fx effectively ameliorated CCl_4_‐induced liver fibrosis by activating Nrf2/HO‐1/GPX4 expression in mice. According to our review of the literature, our study is the first to demonstrate that the hepatoprotective effects of Fx are mainly related to the modulation of the Nrf2/HO‐1/GPX4‐mediated ferroptosis pathway.

The results of this study demonstrate that L‐Fx was more effective in enhancing biochemical markers associated with liver fibrosis, such as AST, ALT, GSH‐ST, and T‐BIL, compared to H‐Fx. This outcome may be due to the excessive activation of the oxidative response feedback mechanism. These findings suggest that the adverse effects of higher dosages surpass the therapeutic advantages, highlighting the necessity for further investigation into the optimal dosage range of this medication. Therefore, we recommend that future preclinical investigations prioritize the development of a dose–response curve to determine the optimal and safest dosage levels. Our research agenda includes conducting additional preclinical and clinical studies aimed at enhancing dose optimization, evaluating the impact of varying doses on therapeutic efficacy and safety, and establishing a comprehensive long‐term monitoring framework to facilitate informed decision‐making. It is noteworthy that during oxidative stress, the activity of antioxidant enzymes is diminished, disrupting the established redox equilibrium (Tan et al. 2018). Nrf2 enters the nucleus from the cytoplasm and binds to antioxidant response elements, thereby promoting the expression of antioxidant proteins. Its downstream protein HO‐1 degrades heme to ferrous iron, whereas its reduced activity triggers ferroptosis (Luo et al. 2022; Cai et al. 2022). GPX4 is an important target that triggers the ferroptosis process. Therefore, the inhibition of downstream SLC7A11 expression inhibits hepatic ferroptosis because of GSH depletion and absence of GPX4 activity (Wang et al. 2017).

Nrf2 is instrumental in mitigating oxidative stress injury. Past research has shown that the activation of the Nrf2 antioxidant signaling pathway can alleviate liver fibrosis (Shin et al. 2021; Sharma et al. 2017). Numerous antioxidants are upregulated when the Nrf2/HO‐1 signaling pathways are activated, thereby protecting the myocardium from damage (Xie et al. 2020). Meanwhile, chronic iron exposure causes iron accumulation and cytosolic reactive oxygen species production. However, via the Nrf2/HO‐1 axis, Nrf2 regulates intracellular iron metabolism, thereby regulating ferroptosis and ultimately altering cell stress (van Raaij et al. 2018). Our western blot analysis revealed a reduced expression of Nrf2 and its downstream proteins in the CCl_4_‐treated group. Notably, treatment with Fx significantly restored the expression levels of these proteins. These results indicate that Fx mitigates oxidative stress injury associated with CCl_4_‐induced liver fibrosis. Furthermore, ferroptosis, characterized by mitochondrial shrinkage, was observed (Tang et al. 2021). Given the critical role of mitochondria in maintaining redox homeostasis and regulating ferroptosis (Wang, Peng, et al. 2020), the present study demonstrates that Fx inhibits ferroptosis in liver fibrosis, thereby attenuating mitochondrial damage induced by CCl_4_; this is consistent with the results of our previous study on CCl_4_‐induced kidney injury (Ding et al. 2025). Research has shown that system Xc^−^ (cystine‐glutamate antiporter) and GPX4 are the two central regulators of ferroptosis and that ferroptosis can be triggered by iron overload, system Xc^−^ inhibition, and GPX4 inactivation (Tang et al. 2021). The GPX4 reduces specific lipid hydroperoxides to lipid alcohols using selenocysteine and glutathione as cofactors (Forcina and Dixon 2019). Thus, when GPX4 activity declines and lipid hydroperoxides accumulate, ferroptosis occurs (Yang and Stockwell 2016). Tsubouchi et al. (2019) discovered that mice with lung fibrosis exhibited decreased GPX4 expression; meanwhile, an interesting observation was that Nrf2 targeted both GPX4 and Xc^−^/xCT, a cystine and glutamate transporter system.

Iron metabolism and transferrin synthesis primarily occur in the liver (Yu et al. 2020). In this process, the transferrin/transferrin receptor system imports iron into the cell and releases it from transferrin in the lysosomes (Forcina and Dixon 2019). It has been shown that immunodepleting transferrin or genetically silencing transferrin receptors can prevent ferroptosis and disrupting the lysosome degradation of the iron storage protein (ferritin) can inhibit ferroptosis (Forcina and Dixon 2019). Ferroptosis is associated with decreased iron uptake caused by TFR1 knockdown. A key component of system Xc^−^, SLC7A11, is inhibited after elastin treatment, leading to GSH depletion and increased ferroptosis (Jiang et al. 2015). The dysfunction of the system Xc^−^/GPX4 pathway may lead to intracellular lipid peroxidation and reactive oxygen species accumulation, ultimately resulting in ferroptosis (Sun et al. 2016). Of note, SLC7A11 has been shown to be a transcriptional target of Nrf2 (Lu et al. 2020). Based on the present study findings, Fx exerts antioxidant properties by upregulating the Nrf2 pathway and GPX4‐mediated ferroptosis to alleviate CCl_4_‐induced murine liver fibrosis. Fx, astaxanthin, lutein, zeaxanthin, and β‐cryptoxanthin are carotenoids known for their anti‐tumor and anti‐inflammatory activities (Pereira et al. 2021). Their chemical structure, characterized by a high number of double bonds, confers significant antioxidant capabilities. The findings of this study suggest that astaxanthin may serve as a promising therapeutic agent for the treatment of hepatic fibrosis, thereby contributing to the high‐value utilization of marine algae and advancing research into novel treatments for hepatic fibrosis.

Nevertheless, this study has certain limitations: (1) it was conducted exclusively using the CCl_4_‐induced liver fibrosis model, necessitating further validation across additional fibrosis models; (2) while regulation of ferroptosis‐related markers by Fx was observed, the study did not employ ferroptosis‐specific inhibitors or genetic tools to confirm pathway specificity; (3) as a preliminary experimental investigation, it did not explore dose–response relationships or conduct long‐term toxicity assessments; (4) the study was constrained by the low purity of the astaxanthin used and the lack of mRNA expression experiments. These limitations should be addressed in future research endeavors. Additionally, the clinical translation of Fx is impeded by several challenges, including low extraction yield, high production costs, limited oral bioavailability, and instability under physiological conditions. Further research is required to develop efficient delivery systems, optimize dosing regimens, and evaluate long‐term toxicity and combination therapy strategies. Future studies should also incorporate both sexes and multiple fibrosis models to enhance translational relevance.

## 5 Conclusions

**FIGURE 6:**
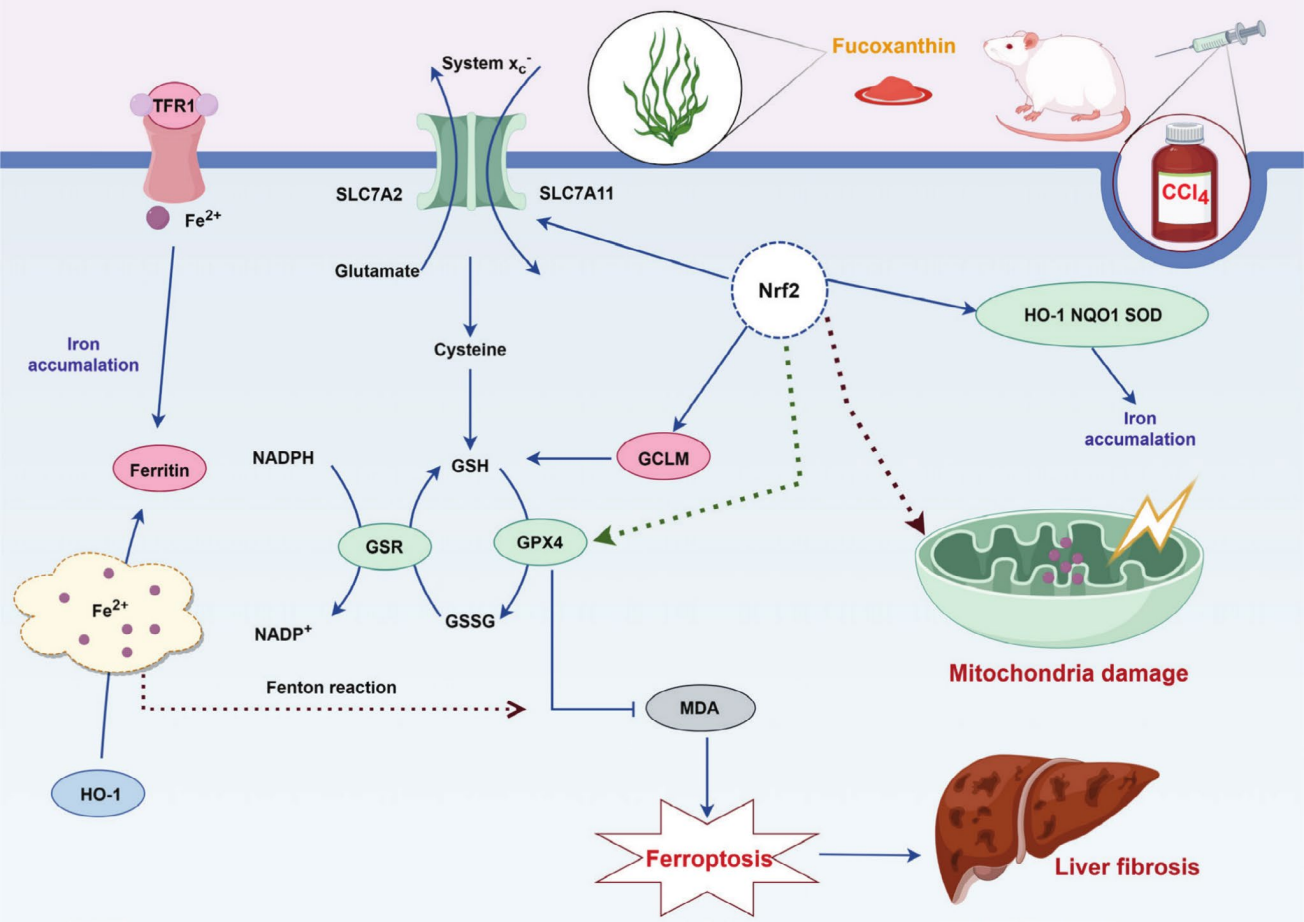
Molecular mechanisms through which Fx alleviated CCl_4_‐induced liver fibrosis in vivo. This figure was drawn by Figdraw (www.figdraw.com).

This study indicates that Fx mitigates CCl_4_‐induced liver fibrosis in mice, likely via activation of the Nrf2/HO‐1/GPX4 axis and inhibition of ferroptosis, reflected by reduced hepatic iron overload, lipid peroxidation, and mitochondrial damage, alongside upregulated GPX4 expression (Figure 6). These results suggest the potential of Fx as a functional food or adjunct therapy for liver fibrosis. Nevertheless, several limitations warrant attention. The exclusive use of the CCl_4_ model restricts generalizability; additional fibrosis models are needed to validate these findings. Ferroptosis involvement was inferred from marker changes without employing specific inhibitors or genetic modulation, limiting mechanistic certainty. The study lacks dose–response analyses and long‐term toxicity evaluations, hindering safety and efficacy assessment. Furthermore, low astaxanthin purity and absence of mRNA‐level data constrain mechanistic insights. In conclusion, while Fx shows promising anti‐fibrotic effects, comprehensive investigations are necessary to overcome current limitations and fully establish its therapeutic utility.

## Author Contributions

**Zhongliang Liu:** conceptualization (equal), data curation (equal), formal analysis (equal), funding acquisition (equal), investigation (equal), writing – original draft (equal). **Jiena Ye:** investigation (equal), methodology (equal), project administration (equal). **Jiachen Xi:** resources (equal), software (equal). **Qingping Li:** validation (equal), visualization (equal). **Yunping Tang:** data curation (equal), formal analysis (equal), validation (equal). **Yizhou Tian:** software (equal), validation (equal), visualization (equal). **Dongxu Wang:** validation (equal), writing – review and editing (equal). **Zuisu Yang:** project administration (equal), resources (equal). **Yaping Ding:** conceptualization (equal), supervision (equal), writing – original draft (equal).

## Conflicts of Interest

The authors declare no conflicts of interest.

## Data Availability

The data that support the findings of this study are available from the corresponding author upon reasonable request.
